# Checkpoint inhibitors plus chemotherapy for first-line treatment of advanced non-small cell lung cancer: a systematic review and meta-analysis of randomized controlled trials

**DOI:** 10.2144/fsoa-2019-0081

**Published:** 2019-09-25

**Authors:** Aung Myint Tun, Kyaw Zin Thein, Wai Lin Thein, Elizabeth Guevara

**Affiliations:** 1 Department of Medicine, Division of Hematology & Oncology, The Brooklyn Hospital Center, Brooklyn, NY 11201, USA; 2 Department of Hematology & Oncology, Texas Tech University Health Sciences Center, Lubbock, TX 79430, USA; 3 University of Medicine 1, Yangon, Myanmar

**Keywords:** advanced non-small-cell lung cancer, checkpoint inhibitors, chemotherapy, first-line therapy, immune-related adverse events, objective response rate, overall survival, progression-free survival, randomized controlled trials, systematic review and meta-analysis

## Abstract

**Background:**

We conducted a meta-analysis to evaluate the efficacy and safety of upfront add-on immunotherapy for advanced non-small cell lung cancers (NSCLC).

**Methods:**

We performed a literature search on first-line chemotherapy ± immunotherapy in NSCLC. We utilized Revman version 5.3 to calculate the estimated pooled hazard ratio for overall survival (OS) and progression-free survival (PFS) and pooled risk ratio for objective response rate (ORR), all-grade and high-grade adverse events with 95% CI.

**Results:**

We analyzed 4322 patients. The pooled hazard ratios for OS, PFS and ORR were 0.74 (95% CI: 0.62–0.88; p = 0.0007), 0.62 (95% CI: 0.57–0.68; p = 0.00001) and 1.51 (95% CI: 1.3–1.74; p = 0.00001), respectively. The pooled risk ratios for all-grade and high-grade adverse events were 1.01 (95% CI: 0.99–1.03; p = 0.27) and 1.17 (95% CI: 1.07–1.28; p = 0.0006), respectively.

**Conclusion:**

Add-on immunotherapy significantly improves PFS, OS and ORR for the first-line treatment of advanced NSCLC with a reasonable safety profile.

## Lay abstract

Lung cancer is the most frequent cancer and is the leading cause of cancer mortality worldwide – more than half of the patients presented at late-stage disease, which is associated with limited survival. To treat cancers, we use immune checkpoint inhibitors that release the brakes on the immune system; thus, the immune cells can kill cancer cells better. Multiple clinical trials have tested the role of immune checkpoint inhibitors combined with chemotherapy for lung cancer treatment. Based on these clinical trials, we conducted a systematic review that showed improvement in outcomes with combined chemotherapy and immunotherapy with acceptable adverse events.

Lung cancer is the most common cancer worldwide, with an estimated incidence of more than 2 million cases and approximately 1.8 million deaths, was also the leading cause of cancer mortality in 2018 [1]. In the USA, it is the second most frequently diagnosed cancer and is currently the leading cause of cancer death in both sexes [2]. There are estimated 228,150 cases of lung and bronchial carcinoma and approximately 142,670 deaths in 2019 in the USA [2]. Non-small cell lung cancer (NSCLC) accounts for approximately 85% of lung cancers [3]. The metastatic disease represents approximately 55% of cases and long-term prognosis remains poor [3].

Subsets of patients with driver mutations and gene rearrangements gain significant benefits from molecularly targeted agents; however, the majority of patients without an identified molecular subtype rely mainly on traditional chemotherapy with modest improvement in survival and quality of life. An increasing in the understanding of the complex interactions between the immune system and cancer has led to the development of immune checkpoint inhibitors, namely monoclonal antibodies directed against programmed death receptor 1 (PD-1), programmed death ligand 1 (PD-L1) and cytotoxic T-cell lymphocyte antigen-4 (CTLA-4) monoclonal antibodies, which promote T-cell activation with subsequent formation of anti-tumor effect, resulting in durable response and improvement in outcome. Single-agent immunotherapy (nivolumab, pembrolizumab and atezolizumab) demonstrated superior overall survival (OS) and better safety profiles compared with chemotherapy in the subsequent management of both squamous and nonsquamous NSCLC [4–7]. However, single-agent immunotherapy in the first-line setting is limited to the small subset of NSCLC patients whose tumors have a PD-L1 tumor proportion score of 50% or more ‘without *EGFR or ALK* genomic tumor aberrations’, for which pembrolizumab is proven to be superior in terms of efficacy and safety profiles [8]. Increasing evidence suggests that combined chemoimmunotherapy can have synergistic anticancer activities through the immunomodulatory effect of checkpoint inhibitors and the immunogenic effect of chemotherapy, such as lowering regulatory T-cell activity and enhancing cross-presentation of tumor antigens [9,10].

Several randomized controlled trials (RCTs) have shown that the addition of immunotherapy to standard chemotherapy improves survival with manageable toxicity profiles. RCTs for the CTLA-4 inhibitor ipilimumab were not included in the analysis owing to separate mechanism of action, lack of OS benefit and different toxicity profiles [11,12]. Therefore, we conducted the meta-analysis of RCTs on PD-1 and PD-L1 inhibitors to evaluate the efficacy and safety of immune checkpoint inhibitors in combination with chemotherapy for the first-line treatment of advanced, metastatic NSCLC.

## Methods

We conducted this systematic review according to the Cochrane Handbook for Systematic Reviews [13] and reported per the Preferred Reporting Items for Systematic Reviews and Meta-Analyses statement.

## Search methods

We conducted literature search in PubMed, EMBASE and SCOPUS databases using the terms ‘immune checkpoint inhibitors and NSCLC’, ‘nivolumab and NSCLC’, ‘pembrolizumab and NSCLC’, ‘atezolizumab and NSCLC’, ‘avelumab and NSCLC’ and ‘durvalumab and NSCLC’. A further search was performed on major oncology conferences throughout January 2019, including those of the American Society of Clinical Oncology, European Society of Medical Oncology and International Association for the Study of Lung Cancer. Clinical trials in English were retrieved and filtered, as mentioned in eligibility criteria.

## Inclusion & exclusion criteria

The search results were narrowed to the following article types: clinical trial, Phase II; clinical trial, Phase III; checkpoint inhibitor plus chemotherapy versus chemotherapy plus placebo; studies that showed survival data and studies conducted for first-line treatment of advanced or metastatic NSCLC.

The exclusion criteria were as follows: review articles, systematic reviews, letter to editor and case reports; preclinical trials, Phase I trials, or nonrandomized trials; immune checkpoint inhibitors in second-line setting; immune checkpoint inhibitors in adjuvant or neoadjuvant settings; duplicates of previous publications on the same population and study of CTLA-4 inhibitor ipilimumab due to lack of OS benefit in combination with standard chemotherapy in the first-line setting [11,12].

## Data extraction & quality assessment

AM Tun and WL Thein collected basic information of individual study and data were extracted independently. Discrepancies and disagreement were resolved through consensus with the third and the fourth reviewers (KZ Thein. and E Guevara). Extracted data include trial name, a surname of the first author, year of publication, study phase, treatment arms, participant characteristics and the number of patients evaluable for analysis. Analysis of hazard ratio (HR) for OS is the primary outcome of the study. Secondary outcomes were pooled progression-free survival (PFS), pooled overall response rate (ORR) and adverse events (AEs). The tool recommended by Cochrane Collaboration (London, UK) identified biases in each study. Biases were classified as selection bias, performance bias, detection bias, attrition bias, reporting bias and others. They are rated as low, high or unclear risk [14].

## Statistical analysis

Review Manager, version 5.3 (Nordic Cochrane Centre; Copenhagen, Denmark) was used for data analyses; p < 0.05 were considered significant and I^2^ >50% is considered substantially heterogeneous [15]. The random-effect model was applied for all analyses due to heterogeneity among studies. We utilized the inverse variance method to analyze PFS and OS data and reported the outcomes as pooled HRs. Analysis of dichotomous outcomes, such as ORR and AEs, were done by the Mantel–Haenszel method and were reported as risk ratios (RRs) with 95% CIs. Subgroup analyses HRs for PFS and OS were conducted based on the degree of PD-L1 expressions (PD-L1 negative; low-PD-L1 expression: PD-L1 tumor proportion score of 1–49% for pembrolizumab trials or PD-L1 expression on 1–49% of tumor cells (TCs) or 1–9% of tumor-infiltrating immune cells (ICs) for atezolizumab trials; and high-PD-L1 expression: PD-L1 of 50% or greater for pembrolizumab trials or PD-L1 expression of 50% or greater on TCs or 10% or higher ICs in atezolizumab trials). We also performed subgroup analyses of PFS based on age (<65 vs ≥65 years), sex (male vs female) and Eastern Cooperative Oncology Group (ECOG) performance status (0 vs 1), and smoking status (current or former vs never). Publication bias was assessed by funnel plots. We did not perform sensitivity analysis since no study notably influences the results.

## Results

### Study selection

**Figure 1.**
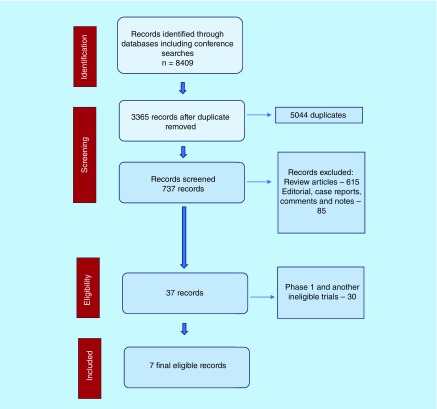
Study flow diagram in accordance with Preferred Reporting Items for Systematic Reviews and Meta-Analyses statement.

We retrieved 8409 potential references, and 5044 duplicates were removed. After application of exclusion criteria as mentioned above, seven RCTs were reviewed for the final analysis. We excluded Keynote 021 trial, which is a Phase II trial with expansion cohort from Phase I trial [16]. The data from IMpower-131, -132 and Checkmate-227 trials were extracted from conference abstracts and presentations. (Figure 1) We incorporated additional data from IMpower-130 trial in the analysis following its publication in July 2019 [17]. Figure 1 demonstrates study selection in accordance with Preferred Reporting Items for Systematic Reviews and Meta-Analyses statement.

### Study characteristics

The characteristics of the included studies are summarized in Table 1. A total of 4322 patients with advanced NSCLC were included in the meta-analysis. Overall, 2991 patients (69%) had nonsquamous NSCLC, among which 152 patients (5%) had *EGFR* or *ALK* alterations. A total of 1331 patients (31%) had squamous NSCLC. The median age of patients ranged from 63 to 65 years; all patients had ECOG performance status score of 0–1 with adequate organ function. All studies were done for the first-line treatment of advanced or metastatic NSCLC utilizing a platinum-based regimen with or without immunotherapy (nivolumab for Checkmate trial, atezolizumab for IMpower trials and pembrolizumab for Keynote trials). Data on the atezolizumab, carboplatin and paclitaxel study arms of IMpower-131 trial (three-arm trial) were not available and not included in the analysis. Another three-arm trial, IMpower-150, had two experimental arms: carboplatin, paclitaxel and atezolizumab (arm A) and carboplatin, paclitaxel, bevacizumab and atezolizumab (arm B) versus carboplatin, paclitaxel and bevacizumab (arm C). We did not include the data comparing between arm A and C because the HR may not reflect the actual effect of add-on immunotherapy (atezolizumab plus chemotherapy vs bevacizumab plus chemotherapy).

**Table 1.** Characteristics of included studies (objective response rate; median progression-free survival; median overall survival; programmed death ligand 1; non-small-cell lung cancer).

Study (year) [Ref.]	Phase	Participants	Patients (n)	Median age (years), study vs control	Intervention	PD-L1 expression	Median follow-up (months)	ORR (%) study arm vs control arm	mPFS (months), study vs control	mOS (months), study vs control
Gandhi *et al.* (2018) [18] Keynote-189	III	Metastatic nonsquamous NSCLC without *EGFR or ALK* mutations	616	65 vs 63.5	Pemetrexed + platin-based drug + pembrolizumab vs pemetrexed + platinum-based drug	≥1%	10.5	47.6 vs 18.9	8.8 vs 4.9	Not reached vs 11.3
Paz-Ares *et al.* (2018) [19] Keynote-407	III	Metastatic squamous NSCLC	559	65	Carboplatin + paclitaxel/nab-paclitaxel + pembrolizumab vs carboplatin + paclitaxel/nab-paclitaxel	Any	7.8	57.9 vs 38.4	6.4 vs 4.8	15.9 vs 11.3
Cappuzzo *et al.* (2018) [17,20] Impower-130	III	Advanced Nonsquamous NSCLC	723 (44 patients had *EGFR* or *ALK* mutations)	64 vs 65	Carboplatin + nabpaclitaxel + atezolizumab vs carboplatin + nabpaclitaxel	Any	NA	49.5 vs 31.9	7 vs 5.5	18.6 vs 13.9
Jotte *et al.* (2018) [21] IMpower-131	III	Metastatic squamous NSCLC	683	65	Carboplatin + nabpaclitaxel + atezolizumab vs carboplatin + nabpaclitaxel	Any	17.1	49 vs 41	6.3 vs 5.6	14 vs 13.9
Papadimitrakopoulou *et al.* (2018) [22] IMpower-132	III	Advanced Nonsquamous NSCLC	578	64 vs 63	Pemetrexed + platin-based drug + atezolizumab vs pemetrexed + platinum-based drug	Any	14.8	47 vs 32	7.6 vs 5.2	18.1 vs 13.6
Socinski *et al.* (2018) [23,24] IMpower-150	III	Metastatic nonsquamous NSCLC	800 (108 patients had *EGFR* or *ALK* alterations)	63	Carboplatin + paclitaxel + bevacizumab + atezolizumab vs Carboplatin + paclitaxel + bevacizumab	Any	15.4 vs 15.5	63.5 vs 48	8.3 vs 6.8	19.2 vs 14.7
Borghaei *et al.* (2018) [25] Checkmate-227	III	Metastatic NSCLC PDL1 <1%	363	64	Chemotherapy + nivolumab vs chemotherapy	<1%	Not available	36.7 vs 23.1	5.6 vs 4.7	pending

NSCLC: Non-small-cell lung cancer; ORR: Objective response rate; PD-L1: Programmed death ligand 1.

Different PD-L1 assay methods were utilized in these studies: 22C3 pharmDx assay (Agilent, CA, USA) in nivolumab and pembrolizumab studies [18,19,26] and SP142 assay (Ventana, Roche, Basel, Switzerland) [23] in atezolizumab studies. Checkmate-227 study comparing platinum-based chemo with or without nivolumab was done on tumors with PD-L1 expression <1% [25].

### Study quality, risk bias & publication bias

**Figure 2.**
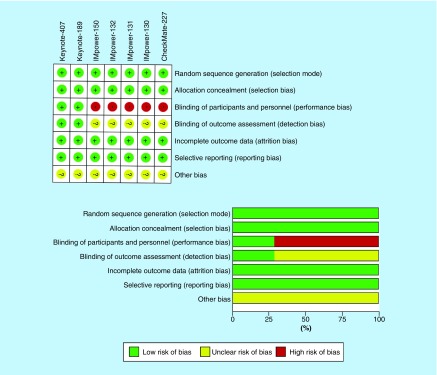
Risk of bias for selected clinical trials.

Risk of bias for each study was evaluated by RevMan 5.3 software (Cochrane) and is illustrated in Figure 2. IMpower-130, -131, -132, -150 and Checkmate-227 were open-label studies that lacked blinding between investigators and participants. Detection bias was unclear for IMpower-130,-131,-132,-150, and Checkmate-227 trials due to lack of blinding. Moreover, all the studies are sponsored by pharmaceutical companies so other biases remained uncertain. Publication bias was not identified in this study.

## Results

### Primary outcome

**Figure 3.**
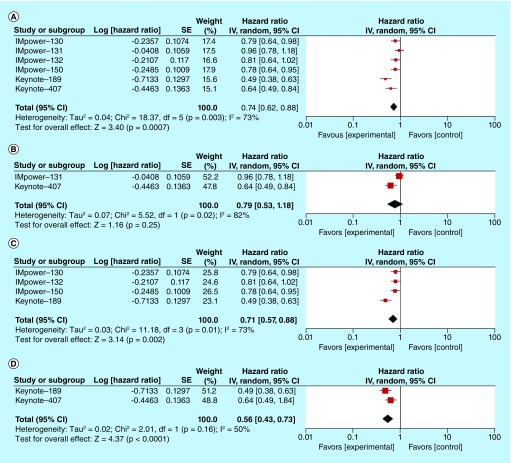

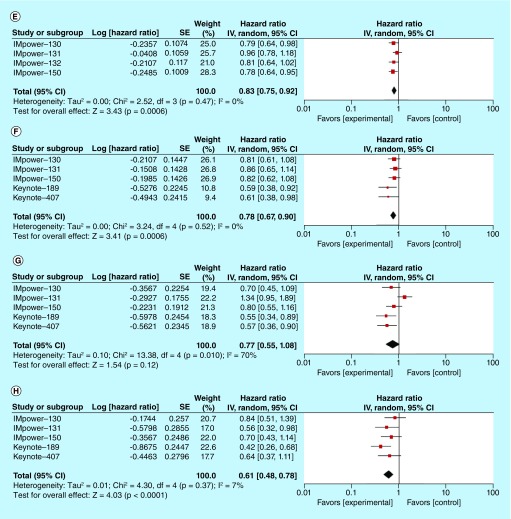
Overall survival analysis in participants treated with first-line chemoimmunotherapy versus chemotherapy alone. **(A)** Pooled HR for OS in patients with advanced NSCLC treated with first-line chemoimmunotherapy. **(B)** Pooled HR for OS in patients with advanced squamous NSCLC treated with first-line chemoimmunotherapy. **(C)** Pooled HR for OS in patients with advanced nonsquamous NSCLC treated with first-line chemoimmunotherapy. **(D)** Pooled HR for OS in patients with advanced NSCLC treated with PD-1 inhibitor (pembrolizumab) in combination with chemotherapy in the first-line setting. **(E)** Pooled HR for OS in patients with advanced NSCLC treated with PD-L1 inhibitor (atezolizumab) in combination with chemotherapy in the first-line setting. **(F)** Pooled HR for OS in PD-L1 negative patients with advanced NSCLC in the first-line setting. **(G)** Pooled HR for OS in PD-L1 low patients with advanced NSCLC in the first-line setting. **(H)** Pooled HR for OS in PD-L1 high patients with advanced NSCLC in the first-line setting. HR: Hazard ratio; NSCLC: Non-small-cell lung cancer; OS: Overall survival; PD-1: Programmed death receptor 1; PD-L1: Programmed death ligand 1.

Median OS ranged from 14 months and is not reached in the Keynote-189 trial in the experimental arms, whereas median OS ranges from 11.3 to 14.7 months in the control arms. The pooled HR for OS was 0.74 (95% CI: 0.62–0.88; p = 0.0007, I^2^ = 73%), and is mentioned in Figure 3A. We performed pooled HR for OS based on histologic subtypes (squamous and nonsquamous) and the type of immunotherapy (PD-1 and PD-L1 monoclonal antibodies). Pooled HRs for OS were 0.79 (95% CI: 0.53–1.18; p = 0.25) for squamous NSCLC (Figure 3B) and 0.71 (95% CI: 0.57–0.88; p = 0.002) for nonsquamous NSCLC (Figure 3C). With regards to different treatment strategies, the pooled HR for PD-1 monoclonal antibody pembrolizumab was 0.56 (95% CI: 0.43–0.73; p = 0001) (Figure 3D) and pooled HR for PD-L1 monoclonal antibody atezolizumab was 0.83 (95% CI 0.75–0.92; p = 0.0006) (Figure 3E). The subgroup analysis was done for different levels of PD-L1 expressions (negative, low and high) and the forest plots for pooled HRs were shown in Figure 3F–H.

### Secondary outcomes

**Figure 4.**
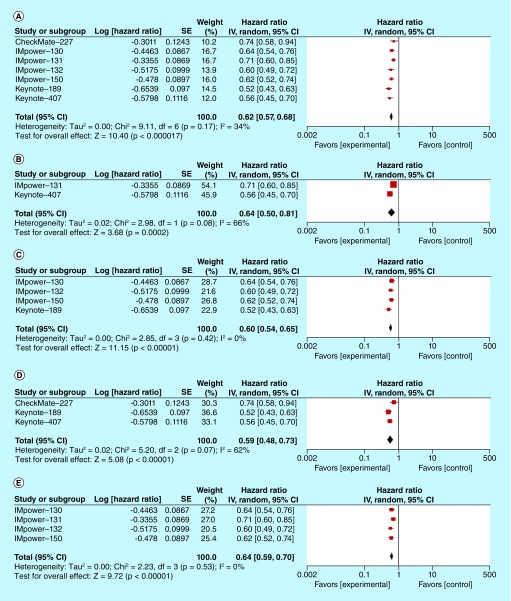

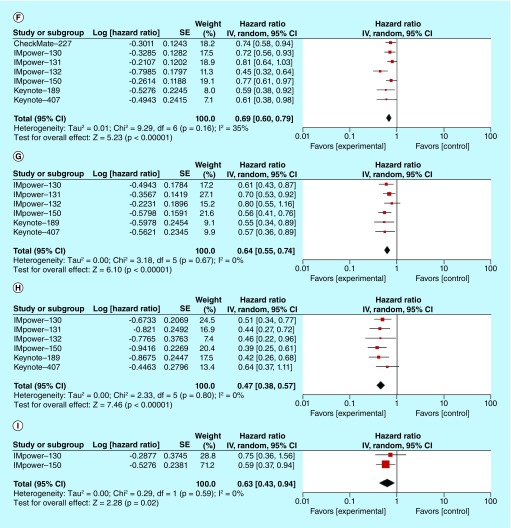
Progression-free survival analysis in participants treated with first-line chemoimmunotherapy versus standard chemotherapy regimen. **(A)** Pooled HR for PFS in patients with advanced NSCLC treated with first-line chemoimmunotherapy. **(B)** Pooled HR for PFS in patients with advanced squamous NSCLC treated with first-line chemoimmunotherapy. **(C)** Pooled HR for PFS in patients with advanced nonsquamous NSCLC treated with first-line chemoimmunotherapy. **(D)** Pooled HR for PFS in patients with advanced NSCLC treated with PD-1 inhibitor (nivolumab or pembrolizumab) in combination with chemotherapy in the first-line setting. **(E)** Pooled HR for PFS in patients with advanced NSCLC treated with PD-L1 inhibitor (atezolizumab) in combination with chemotherapy in the first-line setting. **(F)** Pooled HR for PFS in PD-L1 negative patients with advanced NSCLC in the first-line setting. **(G)** Pooled HR for PFS in PD-L1 low patients with advanced NSCLC in the first-line setting. **(H)** Pooled HR for PFS in PD-L1 high patients with advanced NSCLC in the first-line setting. **(I)** Pooled HR for PFS in patients with *EGFR* and *ALK* mutated advanced NSCLC treated with atezolizumab. HR: Hazard ratio; NSCLC: Non-small-cell lung cancer; OS: Overall survival; PD-1: Programmed death receptor 1; PD-L1: Programmed death ligand 1; PFS: Progression-free survival.

Median PFS ranged from 5.6 to 8.8 months in the study arms, while the control arms ranged from 4.7 to 6.8 months. The pooled HR for PFS was 0.62 (95% CI: 0.57–0.68; p = 0.00001) (Figure 4A). Heterogeneity was present with an I^2^ value of 34%. In addition, we performed pooled HR for PFS based on histologic subtypes (squamous and nonsquamous) and the type of immunotherapy (PD-1 and PD-L1 monoclonal antibodies). Pooled HR for squamous NSCLC and nonsquamous NSCLC were 0.64 (95% CI: 0.50–0.81; p = 0.0002) and 0.6 (95% CI: 0.54–0.65; p = 0.00001) (Figure 4B & C, respectively). The pooled HR for PD-1 monoclonal antibodies nivolumab and pembrolizumab was 0.59 (95% CI: 0.48–0.73; p = 00001) (Figure 4D) and the pooled HR for PD-L1 monoclonal antibody atezolizumab was 0.64 (95% CI: 0.59–0.70; p = 0.00001) (Figure 4E). Subgroup analyses of HRs for PFS based on the degree of PD-L1 expressions were performed and depicted in the Figure 4F–H. The addition of immune checkpoint inhibitor benefited across different levels of PD-L1 expressions. Moreover, subgroup analyses for PFS based on age, sex, ECOG performance status, smoking history and degree of PD-L1 expression (negative, low or high) were summarized in Table 2. IMpower-130 and -150 trials included patients with *EGFR* and *ALK* alterations [17,24]. The pooled HR for PFS in this patient population was 0.63 (95% CI: 0.43–0.94; p = 0.02, I^2^ = 0%), favoring patients treated with atezolizumab (Figure 4).

**Table 2.** Subgroup analyses of pooled hazard ratios for progression-free survival.

Subgroups	Studies (n)	Pooled HR (95% CI)	I^2^ (%)	p-value
Age <65 years	4	0.59 (0.38–0.84)	66%	0.0001
Age ≥65 years	4	0.65 (0.56–0.75)	0%	0.00001
Male	4	0.69 (0.62–0.78)	0%	0.00001
Female	4	0.48 (0.33–0.70)	70%	0.0001
ECOG PS 0	4	0.59 (0.49–0.71)	0%	0.0001
ECOG PS 1	4	0.66 (0.58–0.74)	0%	0.00001
Smoking (current or former)	3	0.64 (0.56–0.73)	19%	0.00001
Smoking (never)	3	0.55 (0.38–0.81)	64%	0.002
PD-L1 <1% (negative)	7	0.69 (0.60–0.79)	35%	0.00001
PD-L1 ≥1–49% (low)	6	0.64 (0.55–0.74)	0%	0.00001
PD-L1 ≥50% (high)	6	0.47 (0.38–0.57)	0%	0.00001

ECOG: Eastern Cooperative Oncology Group; HR: Hazard ratio; PD-L1: Programmed death ligand 1.

**Figure 5.**
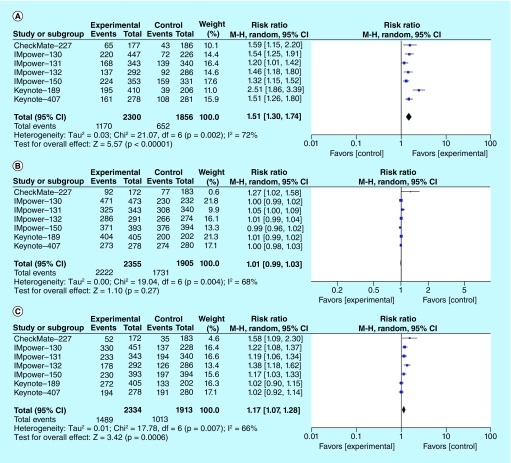
Pooled risk ratio for objective response rate, all-grade adverse events, and high-grade adverse events in patients with NSCLC receiving chemoimmunotherapy versus chemotherapy. **(A)** Pooled RR for ORR in patients with advanced NSCLC treated with first-line chemoimmunotherapy. **(B)** Pooled RR for all-grade AEs in patients with advanced NSCLC treated with first-line chemoimmunotherapy. **(C)** Pooled RR for high-grade AEs in patients with advanced NSCLC treated with first-line chemoimmunotherapy. AE: Adverse event; NSCLC: Non-small-cell lung cancer; ORR: Objective response rate; RR: Risk ratio.

The RR for ORR by random-effect model was 1.51 (95% CI: 1.3–1.74; p = 0.00001, I^2^ = 72%), benefiting the chemoimmunotherapy group (Figure 5A). Higher rates of high-grade (grade 3 or higher) AEs were noted with the addition of immunotherapy in experimental arms, but no significant difference in rates of all-grade AEs was observed. The pooled RR for all-grade AEs (Figure 5B) and high-grade AEs (Figure 5C) were 1.01 (95% CI: 0.99–1.03; p = 0.27, I^2^ = 68%) and 1.17 (95% CI: 1.07–1.28; p = 0.0006, I^2^ = 66%), respectively. Specific immune-related AEs that are associated with statistically significant increased risk, with addition of immune checkpoint inhibitors, include hypothyroidism, hyperthyroidism, pneumonitis, colitis, hepatitis, hypophysitis, infusion reaction and rash. Pooled RRs for specific immune-related AEs are described in Table 3.

**Table 3.** Pooled risk ratios for specific immune-related all-grade adverse events.

Adverse events	Studies (n)	Chemoimmunotherapy		Chemotherapy		Pooled RR (95% CI)	I^2^ (%)	p-value
		Events	Total	Events	Total			
Hypothyroidism	6	226	2174	35	1716	5.18 (2.89–9.27)	51	0.00001
Hyperthyroidism	6	92	2174	18	1716	3.73 (1.70–8.19)	50	0.001
Colitis	6	41	2174	7	1716	3.46 (1.60–7.47)	0	0.002
Pneumonitis	6	117	2174	30	1716	2.92 (1.95–4.37)	0	0.00001
Hepatitis	6	135	2174	50	1716	2.41 (1.27–4.60)	52	0.007
Diabetes	4	10	1605	3	1162	1.77 (0.56–5.59)	0	0.33
Pancreatitis	4	14	1562	3	1102	2.35 (0.75–7.41)	0	0.14
Severe skin reaction	4	21	1367	9	1150	1.63 (0.68–3.88)	12	0.27
Infusion reaction	4	28	1308	10	1090	2.11 (1.02–4.37)	0	0.04
Adrenal insufficiency	3	10	1271	4	828	1.12 (0.23–5.39)	23	0.89
Rash	3	258	1018	149	1002	1.68 (1.13–2.50)	78	0.01
Hypophysitis	3	9	1076	0	876	5.57 (1.01–30.76)	0	0.05
Nephritis	3	12	1076	2	876	2.57 (0.62–10.59)	0	0.19
Meningoencephalitis	2	5	866	0	626	4.13 (0.50–34.37)	0	0.19
Myositis	2	3	798	1	596	1.81 (0.27–12.29)	0	0.55
Thyroiditis	2	4	683	0	482	3.45 (0.39–30.27)	0	0.26
Ocular inflammatory toxicity	1	3	393	0	394	7.02 (0.36–135.41)	NA	0.2
Encephalitis	1	1	393	0	394	3.01 (0.12–73.60)	NA	0.5
Autoimmune hemolytic anemia	1	1	393	1	394	1 (0.06–15.97)	NA	1
Vasculitis	1	1	393	1	394	1 (0.06–15.97)	NA	1

RR: Risk ratio.

## Discussion

Multiple randomized clinical trials have been conducted to identify the optimal chemoimmunotherapy treatment strategy for advanced NSCLC patients. Chen *et al.* conducted pooled efficacy and safety analyses of immune checkpoint inhibitors in NSCLC patients as the first-line treatment option [27]. The study showed statistically improved PFS, OS but not ORR. It is important to note that the analysis also included studies that compare immune checkpoint inhibitors against chemotherapy. We excluded these studies in our review [27]. The meta-analysis done by Xu *et al.* showed improvement in PFS but not OS in first-line treatment of NSCLC [28]. The study mainly includes Phase I trials [28]. Analysis by Shen *et al.* showed improvement in PFS, OS and ORR [29]. A recent meta-analysis by Addeo *et al.* [30] incorporated additional Phase III studies (IMpower-130, -131, -132 and Checkmate-227 studies) that showed significantly prolonged PFS and OS with the addition of immune checkpoint inhibitor [30]. In addition to PFS and OS analyses of *EGFR* and *ALK* wild-type patient population, we analyzed pooled HR for PFS in patients with *EGFR* and *ALK* alterations (Figure 4I) that showed statistically significant PFS benefit. However, this benefit is mainly driven by the IMpower-150 trial, which utilized bevacizumab plus chemotherapy as a backbone regimen. We also performed a comprehensive review of safety profiles. Our study met the primary end point of significantly improved OS. The study also revealed statistically significant improvement in PFS and ORR.

Pooled analysis for OS based on histologic subtypes favored both histologic subtypes (both squamous NSCLC and nonsquamous NSCLC) but was not statistically significant for the squamous NSCLC subtype. A longer follow-up of the IMpower-131 trial may change this result. Besides, we identified substantial heterogeneity with I^2^ of 82% among squamous NSCLC studies with the favorable outcome being driven by the pembrolizumab study (Figure 3B). The pooled HRs for PFS showed statistically significant benefits for both squamous and nonsquamous NSCLC. Moreover, statistically significant OS and PFS benefits were seen on separate analyses of PD-1 and PD-L1 monoclonal antibodies.

Subgroup analyses of both OS and PFS based on the degree of PD-L1 expressions yielded the statistically significant OS and PFS benefits across different levels of PD-L1 expressions, except pooled HR for OS in patients with low PD-L1 expression (HR: 0.77; 95% CI: 0.55–1.08; p = 0.12). We noted substantial heterogeneity in this analysis. The HRs from the Keynote trials contributed more to the survival benefit in low PD-L1 subgroup. In addition, studies utilized different PD-L1 assay methods, further complicating the picture. Nonetheless, subgroup analyses should be interpreted with caution since they are observational by nature and are not based on randomized comparisons. There are significant false positive and false negative findings which could be misleading [13].

Immune checkpoint inhibitors showed relatively tolerable safety profiles. There was no statistically significant increase in rates of all-grade AEs, but a slight increase in high-grade (grade 3 or higher) AEs with the addition of immunotherapy.

There are several limitations to our review. The PD-L1 assay methods are not consistent across different studies; in the atezolizumab studies, PD-L1 immunohistochemistry is read on both TCs and tumor-infiltrating ICs [20,21,23]. However, trials of nivolumab and pembrolizumab applied PD-L1 expression only on TCs [18,19,25]. Moreover, we require longer follow-up and more mature data from the trials, which may change the overall efficacy and safety in the future. At last, our analysis is not designed to identify the optimal combination strategy, but to prove the impact of add-on immunotherapy to chemotherapy in patients with advanced NSCLC.

## Conclusion

Overall, our meta-analysis suggests that the addition of immune checkpoint inhibitor to standard chemotherapy benefited OS and PFS, including tumors with *EGFR* and *ALK* alterations in first-line, advanced, metastatic NSCLC. The analysis also showed statistically significant improvement in ORR in the overall patient population with the addition of immune checkpoint inhibitor. The combined regimen had a slight increase in high-grade AEs without a statistically significant increase in rates of all-grade AEs.

## Future perspective

It is clear that checkpoint blockade, along with cytotoxic chemotherapy, provides additional therapeutic benefit. Despite successful incorporation of PD-1 and PD-L1 inhibitors into the management of metastatic lung cancer, achieving durable remission remains a challenge. Understanding of complex tumor microenvironments and tumor-IC interaction plays a crucial role in the development of novel therapeutic strategies. Combination strategies utilizing multiple immune-checkpoint blockades is an attractive option and have been implemented in the Checkmate-227 trial [26]. Further strategies may include PD-1 blockade in combination with concurrent activation of the immune system, such as vaccination and use of stimulating antibodies. A greater understanding of molecular medicine along with molecular subclassification of this heterogeneous disease and identification of reliable biomarkers to tailor an optimal treatment strategy is crucial in the era of precision oncology. Additional basic science and clinical research will likely help us to understand more about molecular biology, develop newer biomarkers and evolve new therapeutic approaches which will ultimately improve long-term outcome.

**Summary points**

- Immune checkpoint blockade plus chemotherapy is proven to be effective in randomized controlled trials (RCTs) for the treatment of lung cancer.
- We conducted a literature search for RCTs in advanced non-small-cell lung cancer (NSCLC); seven RCTs with a total of 4322 patients were included in the study.
- The study population comprises nonsquamous NSCLC, 69% (5% had *EGFR* or *ALK* alterations) and squamous NSCLC, 31%.
- The analysis showed that the addition of an immune checkpoint inhibitor lead to improvement in overall survival, progression-free survival and objective response rate.
- Addition of atezolizumab to standard therapeutic regimen improved progression-free survival in cohorts of patients with *EGFR* and *ALK* altered nonsquamous NSCLC.
- Subgroup analysis failed to show OS benefit from add-on immunotherapy in patients with squamous NSCLC that expresses low PD-L1.
- The study showed slightly higher rates of high-grade (grade 3 or higher) AEs, but no significant difference in rates of all-grade AEs with addition of immune checkpoint inhibitor.
- We identified substantial heterogeneity among studies. The programmed death ligand 1 assay methods are not consistent across different studies.
- Long-term follow-up is required for mature data.
- Increasing understanding of molecular biology and the development of newer biomarkers and novel therapeutic approaches will improve outcome in this patient population.
